# Hypercalcemia in a male-to-female transgender patient after body contouring injections: a case report

**DOI:** 10.1186/1752-1947-8-71

**Published:** 2014-02-26

**Authors:** Koppany Visnyei, Maria Samuel, Laura Heacock, Jose A Cortes

**Affiliations:** 1 Department of Internal Medicine, Beth Israel Medical Center, Albert Einstein College of Medicine, 1st Ave at 17th St, Baird Hall, 20th Floor, New York, NY 1000, USA

**Keywords:** Lipogranuloma, PTH-independent, Filler

## Abstract

**Introduction:**

Body contouring injections by non-licensed providers are frequently sought out by a subset of the male-to-female transgender community. Although short-term side effects such as pulmonary embolism and injection site infection are well known, long-term consequences of such practices are less well studied.

**Case presentation:**

Here we describe the case of a 40-year-old African American male-to-female transgender patient who presented to our institution with hypercalcemia and acute renal failure secondary to body contouring injections with industrial strength silicone by non-licensed providers, a decade prior to her visit. Work-up revealed an extensive granulomatous inflammatory process in the injection area resulting in electrolyte abnormalities and kidney injury. The patient’s lab results and symptoms responded well to long-term corticosteroid treatment and correlated with treatment adherence.

**Conclusion:**

Affected patients can sometimes present with unusual clinical symptoms many years after silicone injections. In a constantly growing transgender community that often utilizes non-licensed providers for silicone injections, the medical community will likely face an increasing number of patients with long-term side effects of such practices. Therefore, it is imperative for physicians to recognize such cases promptly and initiate potentially life-saving treatment.

## Introduction

Injections with various fillers, including mineral and organic oils like paraffin, petrolatum, camphor oil, silicone and vegetable oils have been practiced for more than a century. The first known injection in modern medical history was performed by an Austrian surgeon, Robert Gersuny in 1899 [1,2]. He injected Vaseline®, a petroleum jelly brand, into the scrotal sack of a patient who underwent orchiectomy due to tuberculous epididymitis. His peer, Eckstein established the use of paraffin as an alternative to petroleum jelly, since it had a higher melting temperature (65°C) and therefore did not soften after the injection. Paraffin was injected in a semi-liquid state after heating, and solidified as it got colder, remaining stable and inert in the human body [1]. Soon other indications followed with both Vaseline® and paraffin, which were used to treat various medical conditions including fistulae, nasal defects and micrognathia, but were also used for wrinkles and body part augmentation, mostly in the breasts and the penis [1]. Such injections remained popular for the first few decades of the 20th century, until long-term side effects became evident in the form of paraffinomas and lipogranulomas. These tumors developed through the dissemination of the foreign material and were extremely difficult to treat [3,4].

Today, an entire century later, we are witnessing a renaissance of body filler injections, albeit with much improved techniques and most importantly, refined, high-tech materials. While such materials seem to enable a relatively safe and side effect poor application, a large number of patients use cheap and unpurified industry-grade products, seemingly due to their financial and accessibility benefits. Additionally, these fillers are often administered by non-licensed providers, exposing patients to further risk of developing health problems. This is a growing problem in distinct subcultures, like the transgender community, where body-contouring is a much sought-after part of body image transformation.

While immediate and short-term complications of such practices are well known and can be diagnosed relatively easily, much less is known about long-term complications, which are also more challenging to recognize [3,5,6]. The major problem is still the development of foreign body granulomatous reactions, and various associated medical conditions. Here we present a case of a transgender patient who presented to our institution with unusual and remarkably serious long-term complications of hypercalcemia and kidney failure after industry-grade silicone injections for body contouring purposes.

## Case presentation

**Figure 1:**
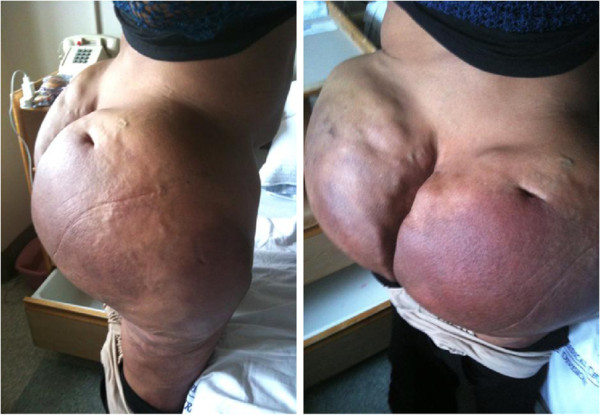
**Physical exam findings in the area of silicone injections.** The patient presented with massive, hardened and painful subcutaneous deposits in the bilateral gluteal and thigh area, one decade after extensive industry-grade silicone injections.

**Figure 2:**
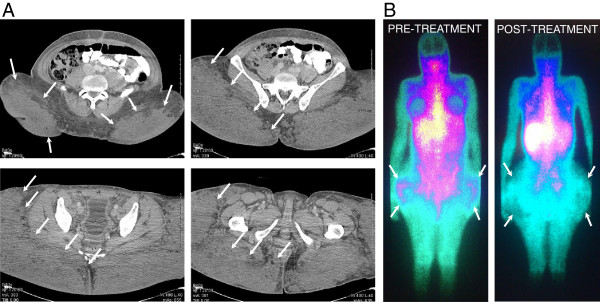
**Imaging data showing extensive grannulomatous disease in the hip region. A** - Axial computed tomography (CT) images showing large amounts of diffusely infiltrating granulomatous tissue (arrows). **B** - Gallium-67 uptake scan showing diffuse tracer uptake in the perigluteal soft tissue, corresponding to the subcutaneous densities on physical exam. Images were taken before (i) and after (ii) initiation of systemic corticosteroid therapy, improving the patient’s condition over time.

**Figure 3:**
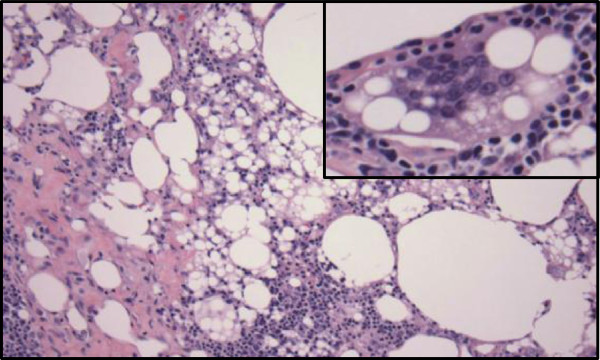
**Groin lymph node biopsy showing benign reactive changes consistent with lipogranuloma.** Note the histiocytes, lipid-containing vacuoles, inflammatory cells and occasional multinucleated giant cells (inset).

A 40-year-old African American male-to-female transgender patient presented with malaise, dysuria, abdominal pain, increased thirst and blurred vision. The patient’s medical history included human immunodeficiency virus (HIV) infection and extensive body contouring injections with industry-grade silicone a decade prior. On physical exam, the patient exhibited massive, hardened, painful and somewhat erythematous bilateral subcutaneous deposits in the gluteal and thigh area (Figure 1). Laboratory studies revealed hypercalcemia (12.3mg/dl, norm: 8.8-10.3mg/dl) and acute kidney injury with elevated creatinine (1.56mg/dl, patient’s baseline 0.70mg/dl, norm: 0.6-1.2mg/dl). The patient had a viral load of <75 copies/ml and a CD4 count of 361 cells/mm^3^ while on Isentress® and Combivir® antiretroviral regimen. She had normal 1,25 dihydroxy-vitamin D (calcitriol) level (47pg/ml; norm: 15-75pg/ml), decreased 25-hydroxyvitamin D (calcidiol) level (16ng/ml; norm: 30-80 ng/ml) and decreased parathyroid hormone (PTH) <2.0pg/ml (norm: 10-60pg/ml).The patient underwent gallium-67 uptake scan that showed diffuse tracer uptake in the perigluteal soft tissue corresponding to the subcutaneous densities on physical exam. Computed tomography (CT) images showed large amounts of diffusely infiltrating granulomatous tissue (Figure 2). Groin lymph node biopsy showed benign reactive changes with histiocytes, lipid-containing vacuoles, inflammatory cells and occasional multinucleated giant cells, consistent with lipogranuloma (Figure 3). The patient’s remaining work-up including chest x-ray, chest CT, anti-nuclear antibody and TSH levels, QuantiFERON®-TB Gold In-Tube Test and fecal ova and parasite test revealed no abnormalities.

Based on the physical, laboratory, imaging and histological findings, the diagnosis of hypercalcemia caused by silicone-induced granulomatous disease was established. In this condition, PTH is typically suppressed by high calcium levels and hypercalcemia occurs due to PTH-independent extrarenal production of 1,25 dihydroxy-vitamin D from 25-hydroxyvitamin D by activated mononuclear cells [7].

**Figure 4:**
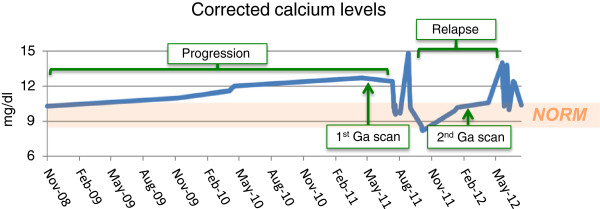
**Corrected serum calcium levels over time.** Note the gradually increasing calcium levels before treatment and the drop of calcium concentrations during therapy. Non-compliance with treatment resulted in repeatedly increasing calcium levels, demonstrating the importance of the long-term use of corticosteroids. Ga, gallium; Norm, normal.

In our patient, additional causes of hypercalcemia, like hyperparathyroidism, myeloma, bony metastases, paraneoplastic syndrome, milk-alkali syndrome, sarcoidosis, tuberculosis, strongyloidiasis or excessive use of vitamin D were ruled out, with laboratory tests and imaging studies as described above. Additionally, the patient did not admit to exogenous vitamin A or estrogen administration and was on continuous antiretroviral therapy without the possibility of immune reconstitution syndrome, which could have all resulted in, or contributed to hypercalcemia [8]. The patient responded well to intravenous hydration, pamidronate and methylprednisone treatment. Her pain subsided, the calcium level and kidney function normalized, and nuclear imaging showed a decreased level of inflammation (Figure 2B). Non-compliance with treatment resulted in increasing calcium levels (up to 18.0mg/dl), demonstrating the importance of the long-term use of corticosteroids. Of note, due to the excessive and diffusely infiltrating nature of her disease, the patient was deemed not to be a surgical candidate (Figure 4).

## Discussion

Injection of organic or inorganic oils has been described to lead to development of a foreign body reaction in the form of a chronic granulomatous inflammation. This happens after an acute inflammatory phase of several months followed by a latent phase that can last several years [9]. The average time to development of first clinical symptoms including sclerodermatous skin changes, tissue hardening, skin hyperpigmentation and development of subcutaneous lumps, nodules and occasional ulcerations, is approximately 6 years [10]. The tumors caused by granulomatous reaction are called oleomas, paraffinomas or siliconomas, depending on the foreign material involved.

Many foreign-body induced tumors have been described in the literature, however the major cause of presentation is almost always of cosmetic nature [4,6]. Our case stands out due to the unusually serious presentation with clinically symptomatic hypercalcemia and renal failure. While the development of calcitriol-related hypercalcemia in granulomatous diseases in general has been well described, it has not been associated with body contouring-induced siliconomas. A potential possibility for the severe electrolyte changes in this particular case might be the unusually large amount of injected material and the large anatomic area involved.

**Figure 5:**
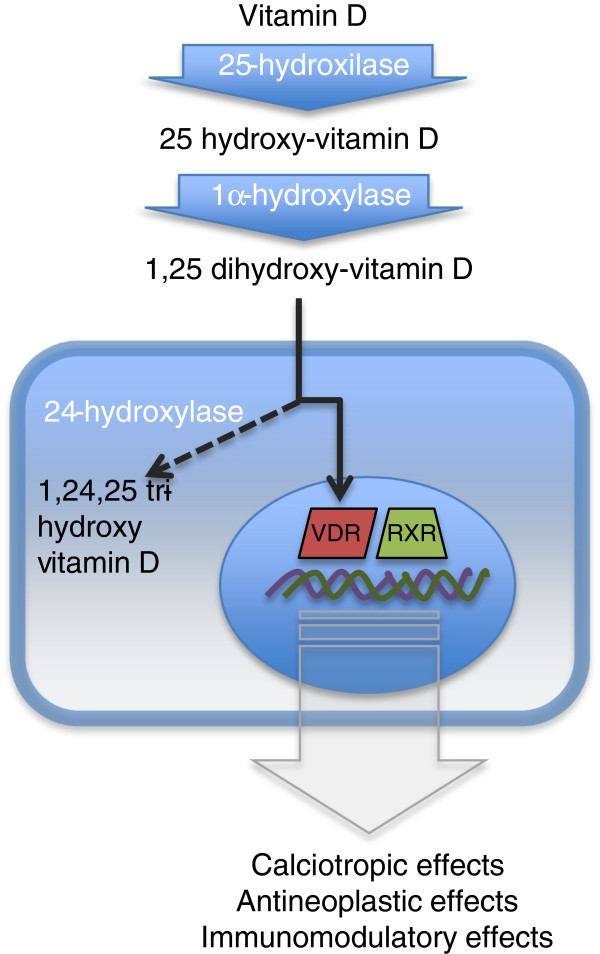
**Production of active vitamin D in renal and immune cells.** Vitamin D3 is initially 25-hydroxylated to the main circulating form of vitamin D, 25-hydroxy vitamin D. This molecule is then 1alpha-hydroxylated to the active form of vitamin D, 1,25-dihydroxy vitamin D. This binds to the vitamin D receptor (VDR) in the nucleus and forms heterodimers with the retinoid X receptor (RXR). The complex acts as a transregulatory complex, controlling transcription of several genes. The enzyme 24-hydroxylase acts as a negative feedback control pathway by synthesizing inactive 1, 24, 25-trihydroxy-vitamin D.

Conversion of inactive vitamin D to its active form requires the enzyme 1-alpha-hydroxylase (also known as cytochrome p450 27B1 or CYP27B1), which is present in renal tubular cells, but can also be found at multiple extrarenal sites, including in immune cells involved in granulomatous diseases, like the alveolar macrophages of patients with sarcoidosis [11,12]. The production of 1,25 dihydroxy-vitamin D is regulated by the inhibition of 1-alpha-hydroxylase activity in response to increased 1,25 dihydroxy-vitamin D levels, and by the intracellular inactivating enzyme 24-hydroxylase. Interestingly, while these inhibiting mechanisms are well established in renal tubular cells, they are much more limited in dendritic cells and macrophages (Figure 5). These latter ones exhibit very little inhibition of 1-alpha-hydroxylase activity, and also have a low concentration of 24-hydroxylase, suggesting a lack of a negative feed-back loop mechanism, resulting in the production of large amounts of 1,25 dihydroxy-vitamin D [13]. It has also been shown that in inflammatory cells, 1,25 dihydroxy-vitamin D production is enhanced by γ-interferon, which is produced by activated lymphocytes and alveolar macrophages in active sarcoidosis [14]. On the other hand, 1,25 dihydroxy-vitamin D has been shown to inhibit activated T helper cells and γ-interferon production. This suggests that the production of 1,25 dihydroxy-vitamin D by macrophages might represent a compensatory mechanism to inhibit the inflammatory process at the site of disease, by limiting T helper cell proliferation and lymphokine production [15]. Regardless of the modulatory effect of 1,25 dihydroxy-vitamin D on inflammatory processes, it is evident that granulomas provide a non-renal source of 1,25 dihydroxy-vitamin D. This has been shown in sarcoid lymph nodes as well as mononuclear cells isolated from tuberculosis patients [16,17], but also in mineral-oil induced paraffinomas, where immunohistochemical studies showed increased 1-alpha-hydroxylase activity in macrophages within of the granulomatous tissue [6].

Elevated 1,25 dihydroxy-vitamin D levels lead to increased intestinal absorption and increased bone resorption of calcium, resulting in hypercalcemia, hypercalcuria, nephrocalcinosis and kidney stones, as well as decreased parathyroid hormone levels. Hypercalcemia is typically worsened by renal insufficiency. It usually varies with calcium intake and can be corrected with dietary restriction of calcium. Exposure to sunlight might also worsen hypercalcemia and can cause seasonal variation of calcium levels [15].

Of note, our patient had normal 1,25 dihydroxy-vitamin D levels upon presentation. One possible explanation for this is the low amount of 25 hydroxy-vitamin D that is available for hydroxylation by 1-alpha-hydroxylase. This would result in lower than expected 1,25 vitamin D levels, even in the presence of increased enzyme activity. Therefore, the relative ratio of 25 hydroxy and 1,25 dihydroxy vitamin D might be more informative than the absolute 1,25 dihydroxy vitamin D level in assessing enzymatic activity in this setting.

Depending on the anatomic location and the extension of the disease, surgical resection can be often performed, either with curative or non-curative, debulking intent. In the case of our patient, surgery was not an option, due to the bare extent of the disease and due to widespread tissue infiltration. Furthermore, the patient declined a figure-altering surgical procedure.

An important observation was that the synthesis of 1,25 dihydroxy-vitamin D in isolated alveolar macrophages of sarcoma patients markedly diminished by the addition of dexamethasone, in a dose-dependent manner [14]. This explains the response of hypercalcemia to steroid treatment in patients with sarcoidosis and other granulomatous diseases. Analogously, foreign material-induced granulomas can be treated conservatively with regionalized corticosteroid injections, especially in the case of smaller lesions. Minocycline, cyclosporine and allopurinol have been used with some success as well [18]. Of note, hydroxychloroquine and ketoconazole have been shown to be effective in treating hypercalcemia in sarcoidosis [19,20]. In contrast to long-term steroids, their milder side effect profiles might make them preferable candidates, however their exact role in treating siliconomas and paraffinomas is still to be explored. In our patient’s case, due to extensive disease, systemic corticosteroid treatment was necessary along with specific calcium lowering management. For the latter, treatment options include reducing calcium intake, elimination of dietary vitamin D supplements, avoidance of sun exposure and if needed, bisphosphonates.

In our case, longitudinally measured calcium levels seemed to have correlated well with corticosteroid treatment and disease activity, as seen on imaging studies. Therefore, it might be a useful tool to monitor disease improvement or progression in patients presenting with extensive disease and severe hypercalcemia.

Renal failure has been extensively described in granulomatous diseases, like sarcoidosis. The main pathophysiological mechanism of kidney involvement seems to be a granulomatous inflammation confined to the tubulointerstitial compartment [21], however interstitial and membranous nephritis without any evidence of granuloma has been described as well [22]. The exact mechanism of development of renal failure in siliconoma or paraffinomas-associated granulomatous disease is still unclear however.

## Conclusion

With a worldwide trend of increasing demand to undergo physique-altering procedures, in the transgender but also other communities, we can expect to see more patients with short and long-term complications of body contouring practices. Long-term complications include localized skin changes and the development of lipogranuloma. However, in case of more extensive disease, hypercalcemia can develop with potentially life-threatening complications. It will be important to recognize these symptoms as possible complications of foreign body injections, and to initiate treatment in a timely manner. Most importantly however, this report would like to stress the importance of informing patients about potential side effects of improper and large-scale body contouring practices by non-licensed providers.

## Consent

Written informed consent was obtained from the patient for publication of this case report and accompanying images. A copy of the written consent is available for review by the Editor-in-Chief of this journal.

## Competing interests

The authors declare that they have no competing interests.

## Authors’ contributions

Our patient was initially admitted under the care of JAC and was followed up in our institution’s outpatient clinic. All authors contributed directly and significantly to the patient’s care during the initial admission and/or various follow-up visits. KV and JAC were major contributors in writing the manuscript. All authors read and approved the final manuscript.
